# Nonlinear Thermal Diffusion and Radiative Stagnation Point Flow of Nanofluid with Viscous Dissipation and Slip Constrains: Keller Box Framework Applications to Micromachines

**DOI:** 10.3390/mi13111839

**Published:** 2022-10-27

**Authors:** Omar T. Bafakeeh, Bilal Ahmad, Skeena Noor, Tasawar Abbas, Sami Ullah Khan, Muhammad Ijaz Khan, Samia Elattar, Sayed M. Eldin, Mowffaq Oreijah, Kamel Guedri

**Affiliations:** 1 Department of Industrial Engineering, Jazan University, Jazan 82822, Saudi Arabia; 2 Department of Mathematics, University of Wah, Wah Cantt 47040, Pakistan; 3 Department of Mathematics, COMSATS University Islamabad, Sahiwal 57000, Pakistan; 4 Department of Mathematics and Statistics, Riphah International University I-14, Islamabad 44000, Pakistan; 5 Department of Mechanical Engineering, Lebanese American University, Beirut 1102, Lebanon; 6 Department of Industrial & Systems Engineering, College of Engineering, Princess Nourah bint Abdulrahman University, P.O. Box 84428, Riyadh 11671, Saudi Arabia; 7 Center of Research, Faculty of Engineering, Future University in Egypt, New Cairo 11835, Egypt; 8 Mechanical Engineering Department, College of Engineering and Islamic Architecture, Umm Al-Qura University, Makkah 21955, Saudi Arabia; 9 Research Unity: Materials, Energy and Renewable Energies, Faculty of Science of Gafsa, University of Gafsa, Gafsa 2100, Tunisia

**Keywords:** thermal flow, nanofluid, stagnation point flow, thermal radiation, viscous dissipation, porous medium

## Abstract

The radiated flow of magnetized viscous fluid subject to the viscous dissipation phenomenon is numerically studied. The radiative phenomenon is addressed with nonlinear relations. Further, analysis is performed by using the slip effects and convective thermal flow constraints. The transformed problem is numerically evaluated using the Keller Box method. The physical parameter effects, such as the magnetic parameter for the velocity profile, Prandtl number, Brownian motion parameter and Biot number for the energy profile and Lewis number, and the thermophoresis parameter for the concentration profile are discussed. The obtained results suggest applications in enhancing the heat transfer phenomenon, thermal system, energy generation, heat transmission devices, power generation, chemical reactions, etc.

## 1. Introduction

The interaction of magnetic forces has significance in engineering applications, such as machine cooling, the lubrication industry, oil pumping, etc. The concept is associated with the impact of electromagnetic body forces on fluid flows. The primary role of magnetic force is in the suction phenomenon, wall motion and blowing. The flow of electrically conducted fluids is usually considered to make the process more effective. The impact of an externally applied magnetic field of a certain strength causes electrical conductivity in the flow of the considered fluids. This is the traditional type of MHD flow mechanism. In sea water, an externally applied magnetic field has much lower thermal conductivity, so an external electrical field is applied to overcome this problem to control the flow. An intersected electric and magnetic field causes the production of a wall-parallel Lorentz force with the objective of varying the procedure of the pressure gradient driven in the boundary layer. Bhatti et al. [1] evaluated the magnetized flow of nanofluids with the enhancement of the solar energy framework. Sabu et al. [2] discussed the particles’ shapes for MHD flow in a rotating flow with the passive control phenomenon. The Williamson nanofluid flow with MHD effects was examined by Song et al. [3]. Jie et al. [4] reported sinusoidal wave flows due to oscillations under a magnetic force impact. Wang et al. [5] performed a bioconvective investigation with the slip phenomenon created by Maxwell liquids.

Nanotechnology is extensively used in industry because nanometer-sized particles provide a number of physical and chemical resources. The term nanofluid was first introduced by Choi [6] in 1995. “Nanofluids” are beneficial for reducing abrasion and reducing in operating components, including pumps and compressors, and result in gasoline savings of more than 6%. It is quite possible that great financial savings can ultimately be made. The appearance of nanofluids is the focal point of research on the sliding of nanofluids in the presence of nanoparticles. Nanofluids consist of particles that are nanometer-sized that slide inside the base fluids. Nanofluids are artificially created colloids containing a base fluid and nanoparticles. Nanoparticles are widely used in improving heat transfer systems, thermal energy, new fuels, hybrid engines, prescription drugs, electricity production and fuel reduction and most cancer remedies. Convection heat transfer fluids, as well as mixtures of oil, ethylene glycol and water, have thermal conductivity, which is important for the constant switching between heat transfer and surface heat transfer. This latest form of liquid was first discovered by increases in thermal conductivity in conventional liquids. The heat transfer mechanism in nanofluid is imprecise, but predictable, as it clearly relies on various additional structures, such as surfactant effects, particle size and particle dispersion of dispersed debris. In addition to progress, nanofluids are regularly used in biomedicine for the identification of tumor cells using nano-scale preparation methods and delivery systems, and breaking blockages in the circulatory system in supply pathways in thallium research. Rasheed et al. [7] modeled the curved surface flow problem with Prandtl nanofluid via convective constraints. Zeeshan [8] reported the entropy-generation significance for viscous nanomaterials with a nonlinear radiation impact. Rasheed et al. [9] evaluated Jeffrey nanofluid due to a vertically moving cylinder with dissipation impact. Zeeshan et al. [10] predicted a rotating surface with a thin film of nanoparticles. Hybrid nanofluid analysis with ethylene glycol as the base liquid was explored by Zeeshan et al. [11]. 

The radiative phenomenon is an interesting mode of heat transfer consisting of the transfer of energy as electromagnetic waves. At high temperatures, the radiation effects are important and have different applications in thermal systems, heat transfer devices, chemical processes, fusion and fission reactions, atomic blasts, nuclear systems, etc. In solar power, gas turbines, aircrafts, space vehicles and missile technology, the application of the radiative phenomenon is also observed. Zhao et al. [12] addressed the features of thermal radiation associated with street canyons. Ramesh et al. [13] presented the decomposition of the thermophoretic phenomenon for carbon nanotubes with radiative analysis. Famakinwa et al. [14] used nonlinear thermal radiation for examining the parallel plate flow of a hybrid nanomaterial with variable features. Ketchate1 et al. [15] observed the onset of nonlinear thermal radiation for aluminum oxide and water-based nanoparticles in porous channels. Some modern developments regarding the fluid flow and nanoparticle concepts are listed in Refs. [16,17,18,19,20,21].

The aim of the current work is to report the stagnation point flow of nanofluid due to a moving surface with magnetic force and porous medium effects. The heat transfer phenomenon is observed by considering the viscous dissipation and radiative phenomenon. The nonlinear thermal radiation relationships are followed, instead of the traditional linear radiation phenomenon. The flow is subject to slip effects. Further, thermal analysis is performed using the convective boundary conditions. For the formulated flow system, the Keller Box numerical scheme is implemented to compute the numerical simulations. It is noted that the Keller Box scheme is widely used in the flow problem; however, numerical simulations for the nanofluid problem with these flow features have not yet been reported. The heat and mass transfer rate and wall shear stress are assessed using a useful resource of plotting the Sherwood Number and Nusselt number relative to several physical elements.

## 2. Mathematical Modeling

A two-dimensional, steady boundary layer incompressible flow of nanofluid is considered over a stretching sheet in the presence of solar radiation (thermal radiation). Flow occurs due to the linear velocity of a surface, which is mathematically written as $u_{w}\left(x\right)=ax$ and is considered through a porous medium. The y-axis of the coordinate system should be perpendicular to the stretching sheet denoted as the *x*-axis. Assume that $\;B_{0}$ is the uniform magnetic field strength, which is applied perpendicularly to the stretching sheet. T  represents the fluid temperature and C represents the concentration. $T_{w}$ is the fluid’s reference temperature, while $T_{\infty }$ is the fluid’s ambient temperature. The effects of viscous dissipation are also incorporated during flow. Under these assumptions, the governing equations after using conservation law are modeled [8,15].

(1) $$\frac{\partial u}{\partial x}+\frac{\partial v}{\partial y}=0,$$

(2) $$\;u\frac{\partial u}{\partial x}+v\frac{\partial v}{\partial y}=u_{\infty }\frac{\partial u_{\infty }}{\partial x}+v_{f}\frac{\partial^{2}u}{\partial y^{2}}-\frac{\sigma_{e}B_{0}{}^{2}}{\rho_{f}}\left(u-u_{\infty }\right)-\frac{\nu }{k_{p}}\left(u_{\infty }-u\right),\;$$

(3) $$u\frac{\partial T}{\partial x}+v\frac{\partial T}{\partial y}=\alpha \frac{\partial^{2}T}{\partial y^{2}}+\frac{v_{f}}{C_{f}}{\left(\frac{\partial u}{\partial y}\right)}^{2}-\frac{1}{{\left(\rho C\right)}_{f}}\left(\frac{\partial q_{r}}{\partial y}\right)+\frac{\sigma_{e}B_{0}^{2}}{{\left(\rho C\right)}_{f}}{\left(u_{\infty }-u\right)}^{2}+\tau \left[D_{B}\frac{\partial T}{\partial y}\frac{\partial C}{\partial y}+\frac{D_{T}}{T_{\infty }}{\left(\frac{\partial T}{\partial y}\right)}^{2}\right]\;$$

(4) $$u\frac{\partial C}{\partial x}+v\frac{\partial C}{\partial y}=D_{B}\frac{\partial^{2}C}{\partial y^{2}}+\frac{D_{T}}{T_{\infty }}\frac{\partial^{2}T}{\partial y^{2}},$$

With boundary conditions given as:

$$y=0\;:u=u_{w}\left(x\right)+L\frac{\partial u}{\partial y}=ax+L\frac{\partial u}{\partial y},\;v=0,-k\frac{\partial T}{\partial y}=h\left(T-T_{w}\right),\;C=C_{w},$$

(5) $$\;y\to \infty \;:u\to u_{\infty }\left(x\right)=bx,\;T\to T_{\infty },\;C\to C_{\infty }.\;$$

The mathematical expression for radiative heat flux is given as follows [13,14]:

(6) $$q_{r}=-\frac{4\sigma^{*}}{3k^{*}}\frac{\partial T^{4}}{\partial y}=-\frac{16\sigma^{*}T^{3}}{3k^{*}}\frac{\partial T}{\partial y},\;$$

Therefore, Equation (3) becomes:

(7) $$u\frac{\partial T}{\partial x}+v\frac{\partial T}{\partial y}=\frac{\partial }{\partial y}\left[\left(\alpha +\frac{16\sigma^{*}T^{3}}{3{\left(\rho C\right)}_{f}k^{*}}\right)\frac{\partial T}{\partial y}\right]+\frac{v_{f}}{C_{f}}{\left(\frac{\partial u}{\partial y}\right)}^{2}+\frac{\sigma_{e}B_{0}^{2}}{{\left(\rho C\right)}_{f}}{\left(u_{\infty }-u\right)}^{2}+\tau \left[D_{B}\frac{\partial T}{\partial y}\frac{\partial C}{\partial y}+\frac{D_{T}}{T_{\infty }}{\left(\frac{\partial T}{\partial y}\right)}^{2}\right]$$

In Equation (7) on the R.H.S., the first term is expressed as [14,15]:

(8) $$\;\alpha \frac{\partial }{\partial y}\left[\left(1+R_{d}{\left(1+\left(\theta_{w}-1\right)\theta \right)}^{3}\right)\frac{\partial T}{\partial y}\right]$$

where $Rd=\frac{16\sigma^{*}T_{\infty }^{3}}{3kk^{*}}$ is the radiation parameter. Equation (7) then becomes:

$$u\frac{\partial T}{\partial x}+v\frac{\partial T}{\partial y}=\alpha \frac{\partial }{\partial y}\left[\left(1+R_{d}{\left(1+\left(\theta_{w}-1\right)\theta \right)}^{3}\right)\frac{\partial T}{\partial y}\right]+\frac{v_{f}}{C_{f}}{\left(\frac{\partial u}{\partial y}\right)}^{2}+\frac{\sigma_{e}B_{0}^{2}}{{\left(\rho C\right)}_{f}}{\left(u_{\infty }-u\right)}^{2}+\tau \left[D_{B}\frac{\partial T}{\partial y}\frac{\partial C}{\partial y}+\frac{D_{T}}{T_{\infty }}{\left(\frac{\partial T}{\partial y}\right)}^{2}\right]$$

By using the similarity transformation

(9) $$\eta =\sqrt{\frac{a}{\upsilon }}y,\;u=axf^{\prime }\left(\eta \right),\;v=-\sqrt{av_{f}}f,\;\theta \left(\eta \right)=\frac{T-T_{\infty }}{T_{w}-T_{\infty }},\;\varphi \left(\eta \right)=\frac{C-C_{\infty }}{C_{w}-C_{\infty }}\;,$$

the continuity equation will be identically satisfied and Equations (2)–(4) can be written as:

(10) $$f^{\prime \prime \prime }+ff^{\prime \prime }-{\left\{f^{\prime }\right\}}^{2}+\lambda^{2}+M\left(\lambda -f^{\prime }\right)-Kf^{\prime }=0\;$$

(11) $$\frac{1}{Pr}\left[\theta^{\prime \prime }\left(1+R_{d}{\left(1+\left(\theta_{w}-1\right)\theta \right)}^{3}\right)+3\theta^{\prime 2}\left(\theta_{w}-1\right)R_{d}{\left(1+\left(\theta_{w}-1\right)\theta \right)}^{2}\right]+f\theta^{\prime }+E_{C}^{*}{f^{\prime \prime }}^{2}+ME_{C}^{*}{\left(\lambda -f^{\prime }\right)}^{2}+N_{b}\theta^{\prime }\phi^{\prime }+N_{t}{\theta^{\prime }}^{2}=0\;$$

(12) $$\phi^{\prime \prime }+Lef\varphi^{\prime }+\frac{N_{t}}{N_{b}}\theta^{\prime \prime }=0$$

With the thermal slip boundary conditions:

(13) $$f\left(\eta \right)=0,\;f^{\prime }\left(\eta \right)=1+\beta f^{\prime \prime }\left(\eta \right),\;\;\theta^{\prime }\left(\eta \right)=-Bi\left(1-\theta \left(\eta \right)\right),\;\;\varphi \left(\eta \right)=1\;\;\;\;\;\mathrm{as}\;\eta =0$$

(14) $$\;\eta \to \infty :f^{\prime }\left(\eta \right)\to \lambda ,\theta \left(\eta \right)\to 0,\varphi \left(\eta \right)\to 0\;$$

The involved physical parameters are defined as:

where $M=\frac{\sigma B_{0}}{\rho_{f}a}$ is the magnetic parameter, $K=\frac{\nu }{k_{p}}$ is the porosity parameter, $\beta =L\sqrt{\frac{a}{v_{f}}}$ represents the slip parameter, $\lambda =\frac{b}{a}$ represents the stretching parameter, $Bi=\frac{h}{k}\sqrt{\frac{v_{f}}{a}}$ is the Biot number, $Nb=\frac{\tau D_{B}\left(C_{w}-C_{\infty }\right)}{v_{f}}$ is the Brownian motion parameter, $Nt=\frac{\tau D_{T}\left(T_{w}-T_{\infty }\right)}{T_{\infty }v_{f}}$ is the thermophoresis parameter, $E_{c}^{*}=\frac{U_{W}^{2}}{C_{p}(T_{w-T_{\infty })}}$ is the local Eckert number, $Le=\frac{v}{D_{B}}$ is the Lewis number and $Pr=\frac{v_{f}}{a}$ is the Prandtl number.

## 3. Keller Box Scheme

The nonlinear coupled system justified via Equations (10)–(12) is numerically solved with the Keller Box scheme. This scheme was used due to its high accuracy. First, the governing problem is reduced into the first-order equations

(15) $$f^{\prime }=u$$

(16) $$u^{\prime }=v$$

(17) $$\theta^{\prime }=p$$

(18) $$\varphi^{\prime }=g$$

Equations (1)–(3) become:

(19) $$v^{\prime }+fv-u^{2}+\lambda^{2}+M\left(\lambda -u\right)-Ku=0$$

(20) $$\frac{1}{Pr}\left[p^{\prime }\left(1+Rd{\left(1+\left(\theta_{w}-1\right)\theta \right)}^{3}\right)+3p^{2}\left(\theta_{w}-1\right)Rd{\left(1+\left(\theta_{w}-1\right)\theta \right)}^{2}\right]+fp+N_{b}pg+N_{t}p^{2}+Ec^{*}v^{2}+MEc^{*}{\left(\lambda -u\right)}^{2}=0$$

(21) $$g^{\prime }+Lefg+\frac{N_{t}}{N_{b}}p^{\prime }=0$$

Therefore, BC becomes:

(22) $$f\left(0\right)=0,\;\;\;u\left(0\right)=1+\beta v,\;\;\;p\left(0\right)=-B_{i}\left[1-\theta \left(0\right)\right],\;\;\;\varphi \left(0\right)=1\;\;\;\;\;\mathrm{at}\;\eta =0$$

(23) u(∞)→λ,   θ(∞)→0,   ϕ(∞)→0   at η→∞

By applying the Keller Box method:

$$\frac{f_{j}-f_{j-1}}{h_{j}}=\frac{u_{j}+u_{j-1}}{2}$$

(24) $$f_{j}-f_{j-1}-\frac{h_{j}}{2}\left(u_{j}+u_{j-1}\right)=0$$

$$\frac{u_{j}-u_{j-1}}{h_{j}}=\frac{v_{j}+v_{j-1}}{2}$$

(25) $$u_{j}-u_{j-1}-\frac{h_{j}}{2}\left(v_{j}+v_{j-1}\right)=0$$

$$\frac{\theta_{j}-\theta_{j-1}}{h_{j}}=\frac{p_{j}+p_{j-1}}{2}$$

(26) $$\theta_{j}-\theta_{j-1}-\frac{h_{j}}{2}\left(p_{j}+p_{j-1}\right)=0$$

$$\frac{\varphi_{j}-\varphi_{j-1}}{h_{j}}=\frac{g_{j}+g_{j-1}}{2}$$

(27) $$\varphi_{j}-\varphi_{j-1}-\frac{h_{j}}{2}\left(g_{j}+g_{j-1}\right)=0$$

Using Equations (10)–(13) in Equations (5)–(7), we get:

(28) $$v_{j}-v_{j-1}+h_{j}[\frac{1}{4}\left(f_{j}+f_{j-1}\right)\left(v_{j}+v_{j-1}\right)-{\left(\frac{u_{j}+u_{j-1}}{2}\right)}^{2}+\lambda^{2}+M\left\{\lambda -\left(\frac{u_{j}+u_{j-1}}{2}\right)\right\}-\frac{K}{2}\{u_{j}+u_{j-1}\}]=0$$

(29) $$\frac{1}{Pr}\left[\left(\frac{p_{j}-p_{j-1}}{h_{j}}\right)\left(1+Rd{\left(1+\left(\theta_{w}-1\right)\left(\frac{\theta_{j}+\theta_{j-1}}{2}\right)\right)}^{3}\right)+3{\left(\frac{p_{j}+p_{j-1}}{2}\right)}^{2}(\theta_{w}-1)Rd{\left(1+\left(\theta_{w}-1\right)\left(\frac{\theta_{j}+\theta_{j-1}}{2}\right)\right)}^{2}\right]+\left(\frac{f_{j}+f_{j-1}}{2}\right)\left(\frac{p_{j}+p_{j-1}}{2}\right)+N_{b}\left(\frac{p_{j}+p_{j-1}}{2}\right)\left(\frac{g_{j}+g_{j-1}}{2}\right)+N_{t}{\left(\frac{p_{j}+p_{j-1}}{2}\right)}^{2}+Ec^{*}{\left(\frac{v_{j}+v_{j-1}}{2}\right)}^{2}+MEc^{*}\left[{\left(\lambda -\left(\frac{u_{j}+u_{j-1}}{2}\right)\right)}^{2}\right]=0$$

(30) $$p_{j}-p_{j-1}+\frac{h_{j}}{\left(1+Rd{\left(1+\left(\theta_{w}-1\right)\left(\frac{\theta_{j}+\theta_{j-1}}{2}\right)\right)}^{3}\right)}\left[\frac{\mathrm{Pr}}{4}\left\{\left(f_{j}+f_{j-1}\right)\left(p_{j}+p_{j-1}\right)+N_{b}\left(p_{j}+p_{j-1}\right)\left(g_{j}+g_{j-1}\right)+N_{t}{\left(p_{j}+p_{j-1}\right)}^{2}+Ec^{*}{\left(v_{j}+v_{j-1}\right)}^{2}+MEc^{*}\left[{\left(2\lambda -\left(u_{j}+u_{j-1}\right)\right)}^{2}\right]\right\}+3{\left(\frac{p_{j}+p_{j-1}}{2}\right)}^{2}(\theta_{w}-1)Rd{\left(1+\left(\theta_{w}-1\right)\left(\frac{\theta_{j}+\theta_{j-1}}{2}\right)\right)}^{2}\right]=0$$

(31) $$g_{j}-g_{j-1}+\frac{h_{j}}{4}Le\left(f_{j}+f_{j-1}\right)\left(g_{j}+g_{j-1}\right)+\frac{N_{t}}{N_{b}}\left(p_{j}-p_{j-1}\right)=0$$

## 4. Newton Method for Linearization

Linearize the system of nonlinear equations:

(32) $$f_{j}^{\left(i+1\right)}=f_{j}^{\left(i\right)}+\delta f_{j}^{\left(i\right)}$$

(33) $$u_{j}^{\left(i+1\right)}=u_{j}^{\left(i\right)}+\delta u_{j}^{\left(i\right)}$$

(34) $$v_{j}^{\left(i+1\right)}=v_{j}^{\left(i\right)}+\delta v_{j}^{\left(i\right)}$$

(35) $$p_{j}^{\left(i+1\right)}=p_{j}^{\left(i\right)}+\delta p_{j}^{\left(i\right)}$$

(36) $$\theta_{j}^{\left(i+1\right)}=\theta_{j}^{\left(i\right)}+\delta \theta_{j}^{\left(i\right)}$$

(37) $$\varphi_{j}^{\left(i+1\right)}=\varphi_{j}^{\left(i\right)}+\delta \varphi_{j}^{\left(i\right)}$$

Therefore, Equation (18) becomes:

(38) $$\delta f_{j}-\delta f_{j-1}-\frac{h_{j}}{2}\left[\delta u_{j}+\delta u_{j-1}\right]={\left(r_{1}\right)}_{j}$$

(39) $$\delta u_{j}-\delta u_{j-1}-\frac{h_{j}}{2}\left[\delta v_{j}+\delta v_{j-1}\right]={\left(r_{2}\right)}_{j}$$

(40) $$\delta \theta_{j}-\delta \theta_{j-1}-\frac{h_{j}}{2}\left[\delta p_{j}+\delta p_{j-1}\right]={\left(r_{3}\right)}_{j}$$

(41) $$\delta \varphi_{j}-\delta \varphi_{j-1}-\frac{h_{j}}{2}\left[\delta g_{j}+\delta g_{j-1}\right]={\left(r_{4}\right)}_{j}$$

where

$${\left(r_{1}\right)}_{j}=f_{j-1}-f_{j}+h_{j}u_{j-\frac{1}{2}}$$

$${\left(r_{2}\right)}_{j}=u_{j-1}-u_{j}+h_{j}v_{j-\frac{1}{2}}$$

$${\left(r_{3}\right)}_{j}=\theta_{j-1}-\theta_{j}+h_{j}p_{j-\frac{1}{2}}$$

$${\left(r_{4}\right)}_{j}=p_{j-1}-p_{j}+h_{j}g_{j-\frac{1}{2}}$$

Equation (14) becomes:

(42) $$\delta v_{j}\left[A_{1}\right]+\delta v_{j-1}\left[A_{2}\right]+\delta f_{j}\left[A_{3}\right]+\delta f_{j-1}\left[A_{4}\right]+\delta u_{j}\left[A_{5}\right]+\delta u_{j-1}\left[A_{6}\right]=r_{5}$$

Equation (15) becomes:

(43) $$\delta p_{j}\left[B_{1}\right]+\delta p_{j-1}\left[B_{2}\right]+\delta f_{j}\left[B_{3}\right]+\delta f_{j-1}\left[B_{4}\right]+\delta g_{j}\left[B_{5}\right]+\delta g_{j-1}\left[B_{6}\right]+\delta v_{j}\left[B_{7}\right]+\delta v_{j-1}\left[B_{8}\right]=r_{6}$$

Equation (17) becomes:

(44) $$\delta g_{j}\left[C_{1}\right]+\delta g_{j-1}\left[C_{2}\right]+\delta f_{j}\left[C_{3}\right]+\delta f_{j-1}\left[C_{4}\right]+\delta p_{j}\left[C_{5}\right]+\delta p_{j-1}\left[C_{6}\right]=r_{7}$$

where

$$A_{1}=1+\frac{h_{j}}{2}f_{j-\frac{1}{2}},\;A_{2}=-1+\frac{h_{j}}{2}f_{j-\frac{1}{2}},A_{3}=A_{4}=\frac{h_{j}}{2}v_{j-\frac{1}{2}},\;A_{5}=A_{6}=-\frac{1}{2}+\frac{M}{2}-\lambda -\frac{K}{2}$$

$$B_{1}=1+\frac{1}{2}f_{j-\frac{1}{2}}+\frac{N_{b}}{2}g_{j-\frac{1}{2}}+N_{t}p_{j-\frac{1}{2}},\;B_{2}=-1+\frac{1}{2}f_{j-\frac{1}{2}}+\frac{N_{b}}{2}g_{j-\frac{1}{2}}+N_{t}p_{j-\frac{1}{2}}$$

$$B_{3}=B_{4}=\frac{1}{2}p_{j-\frac{1}{2}}+ME_{c}^{*}(f_{j-\frac{1}{2}}+\lambda ),\;B_{5}=B_{6}=\frac{N_{b}}{2}p_{j-\frac{1}{2}},\;B_{7}=B_{8}=E_{c}^{*}p_{v-\frac{1}{2}}$$

$$C_{1}=1+\frac{h_{j}}{2}Lef_{j-\frac{1}{2}},\;C_{2}=1-\frac{h_{j}}{2}Lef_{j-\frac{1}{2}},\;C_{3}=C_{4}=\frac{h_{j}}{2}Leg_{j-\frac{1}{2}},\;C_{5}=C_{6}=\frac{N_{t}}{N_{b}}$$

and

$$r_{5}=v_{j-1}-v_{j}-h_{j}[\left(f_{j-\frac{1}{2}}\right)\left(v_{j-\frac{1}{2}}\right)-{\left(u_{j-\frac{1}{2}}\right)}^{2}-\lambda^{2}-M\left\{\lambda^{2}+{\left(u_{j-\frac{1}{2}}\right)}^{2}-\lambda \left(u_{j}+u_{j-1}\right)\right\}+\frac{K}{2}\left(u_{j}+u_{j-1}\right)$$

$$r_{6}=p_{j-1}-p_{j}+\;\frac{Pr}{1+Rd{\left(1+\left(\theta_{w}-1\right)\left(\frac{\theta_{j}+\theta_{j-1}}{2}\right)\right)}^{3}}\left[\left(p_{j-\frac{1}{2}}\right)\left(f_{j-\frac{1}{2}}\right)+E_{c}^{*}{\left(v_{j-\frac{1}{2}}\right)}^{2}+ME_{c}^{*}\left\{\lambda^{2}+{\left(f_{j-\frac{1}{2}}\right)}^{2}+\lambda \left(f_{j}+f_{j-1}\right)\right\}+\frac{N_{b}}{2}\left(f_{j-\frac{1}{2}}\right)\left(g_{j-\frac{1}{2}}\right)+N_{t}{\left(p_{j-\frac{1}{2}}\right)}^{2}\right]+3\left(\theta_{w}-1\right)Rd{\left(1+\left(\theta_{w}-1\right)\left(\frac{\theta_{j}+\theta_{j-1}}{2}\right)\right)}^{2}{\left(p_{j-\frac{1}{2}}\right)}^{2}$$

$$r_{7}=g_{j-1}-g_{j}-\frac{h_{j}}{2}Le\left(g_{j-\frac{1}{2}}\right)\left(f_{j-\frac{1}{2}}\right)-\frac{N_{t}}{N_{b}}\left(p_{j}-p_{j-1}\right)$$

$$f\left(0\right)=0,\;\;\;u\left(0\right)=1+\beta v,\;\;\;p\left(0\right)=-B_{i}\left[1-\theta \left(0\right)\right],\;\;\;\varphi \left(0\right)=1\;\;\;\;\;\;\;\mathrm{at}\;\eta =0$$

(45) u(∞)→λ,   θ(∞)→0,   ϕ(∞)→0    at η→∞

## 5. Computational Method

In this section, the arising PDEs that are nonlinear along with the BCs are solved by using the Keller Box method. For this, non-linear equations are first converted into ordinary differential equations of the first order. The conversion process is carried out by using the following transformation:

$$\left(y_{1},y_{2},y_{3},y_{4},y_{5},y_{6},y_{7},y_{8},y_{9},y_{10}\right)=\left(f,f^{\prime },f^{\prime \prime },f^{\prime \prime \prime }\theta ,\theta^{\prime },\theta^{\prime \prime },\emptyset ,\emptyset^{\prime },\emptyset^{\prime \prime }\right)$$

$$\left(\begin{array}{c} {y^{\prime }}_{1}\\ {y^{\prime }}_{2}\\ {y^{\prime }}_{3}\\ {y^{\prime }}_{4}\\ {y^{\prime }}_{5}\\ {y^{\prime }}_{6}\\ {y^{\prime }}_{7}\\ {y^{\prime }}_{8} \end{array}\right)=\left(\begin{array}{c} 1\\ y_{3}\\ y_{4}\\ \left[y_{3}^{2}-y_{2}y_{4}-\lambda^{2}-M\left(\lambda -y_{3}\right)-Ky_{4}\right]\\ y_{6}\\ \frac{-1}{1+Rd{\left(1+zy_{4}\right)}^{3}}\left[\mathrm{Pr}\left\{y_{1}y_{5}+{\mathrm{Nby}}_{5}y_{6}+\mathrm{Nt}{\left(y_{5}\right)}^{2}+Ec^{*}y_{3}^{2}+MEc{\left(\lambda -y_{2}\right)}^{2}\right\}+3y_{5}^{2}zRd{\left(1+zy_{4}\right)}^{2}\right]\\ y_{8}\\ -\left(Ley_{2}y_{8}+\frac{N_{t}}{N_{b}}{y^{\prime }}_{6}\right) \end{array}\right)$$

(46) $$\left(\begin{array}{c} y_{1}\left(0\right)\\ y_{2}\left(0\right)\\ y_{3}\left(0\right)\\ y_{4}\left(0\right)\\ y_{5}\left(0\right)\\ y_{6}\left(0\right)\\ y_{7}\left(0\right) \end{array}\right)=\left(\begin{array}{c} u_{1}\\ 1+\beta f^{\prime \prime }\\ \lambda \\ u_{2}\\ -Bi\left(1-y_{4}\right)\\ 1\\ u_{3} \end{array}\right).$$

The above system of first-order ODEs and corresponding ICs is resolved using the Keller Box method. The corresponding values of the mysterious ICs $u_{1},u_{2}\;and\;u_{3}$ are considered by Newton’s approach to BCs $f^{\prime }\left(\eta \right)\to \lambda ,\theta \left(\eta \right)\to 0,\phi \left(\eta \right)\to 0\;as\;n\to \infty$ and the boundary conditions are satisfied. The calculations were conducted using the mathematical software MATLAB.

## 6. Results and Discussion

**Figure 1:**
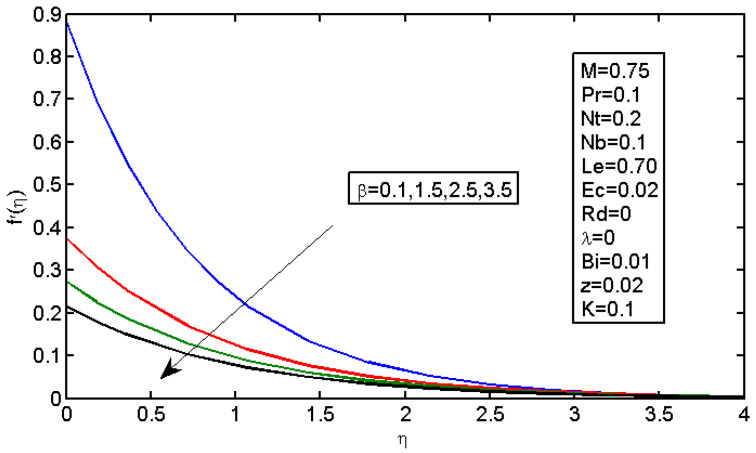
Impact of β on the velocity profile.

**Figure 2:**
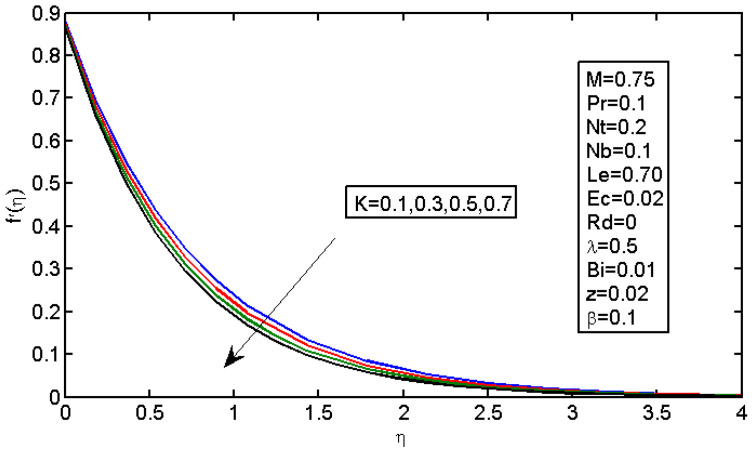
Impact of K on the velocity profile.

**Figure 3:**
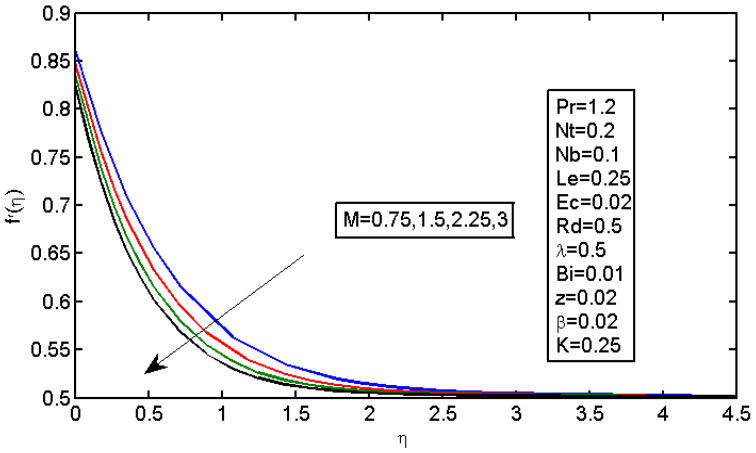
Impact of M on the velocity profile.

**Figure 4:**
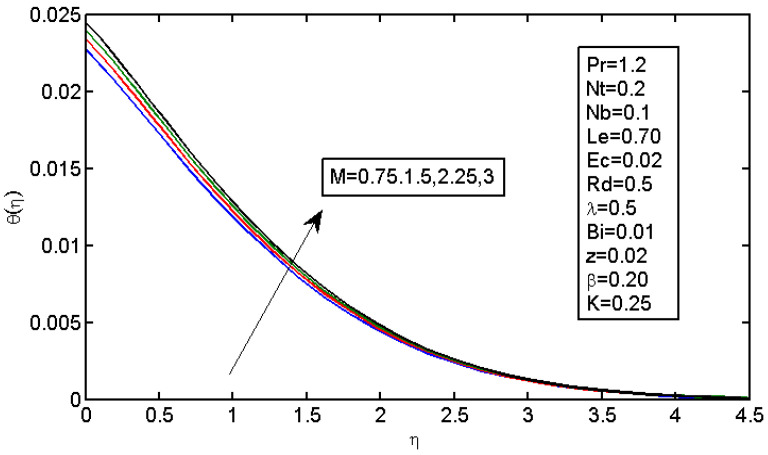
Impact of M on the temperature profile.

**Figure 5:**
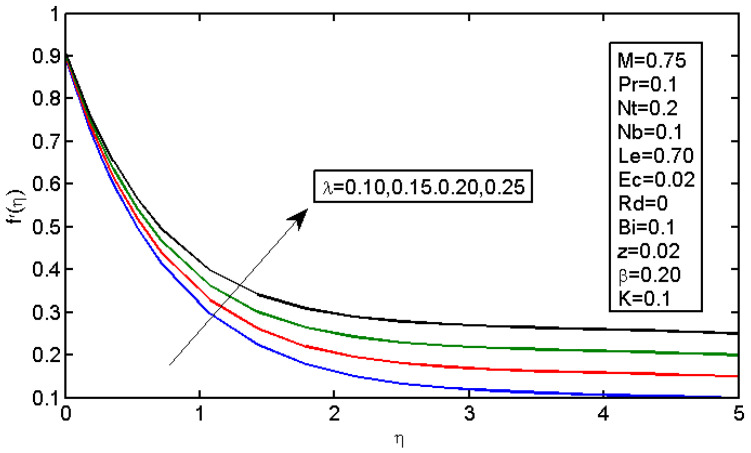
Impact of λ on the velocity profile.

**Figure 6:**
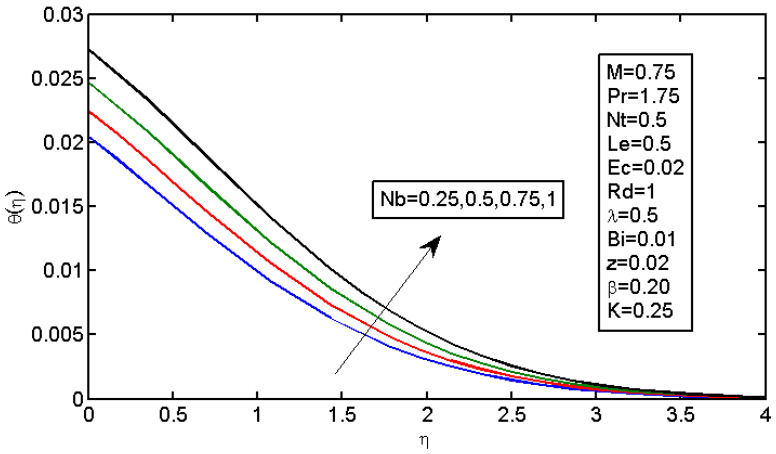
Impact of $N_{b}$ on the temperature profile.

**Figure 7:**
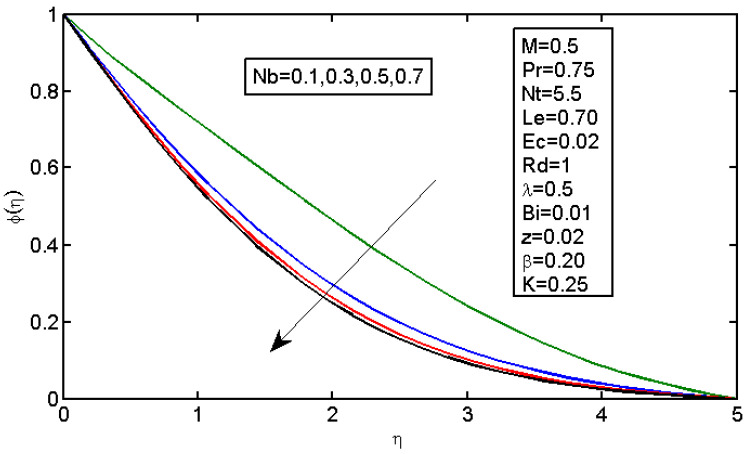
Impact of $N_{b}$ on the concentration profile.

**Figure 8:**
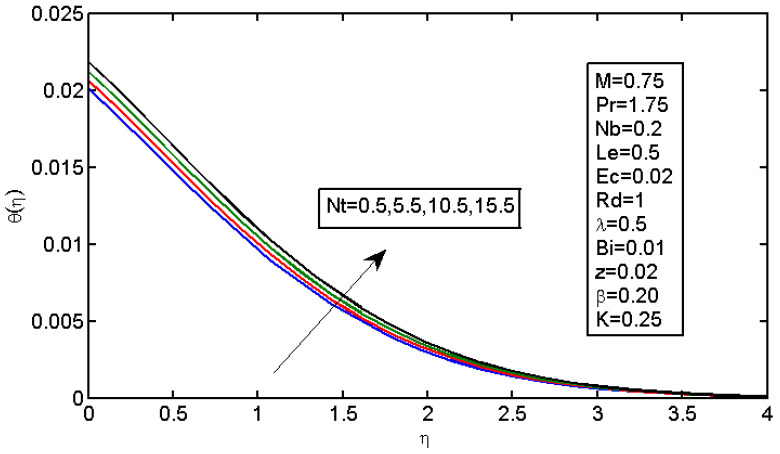
Impact of $N_{t}$ on the temperature profile.

**Figure 9:**
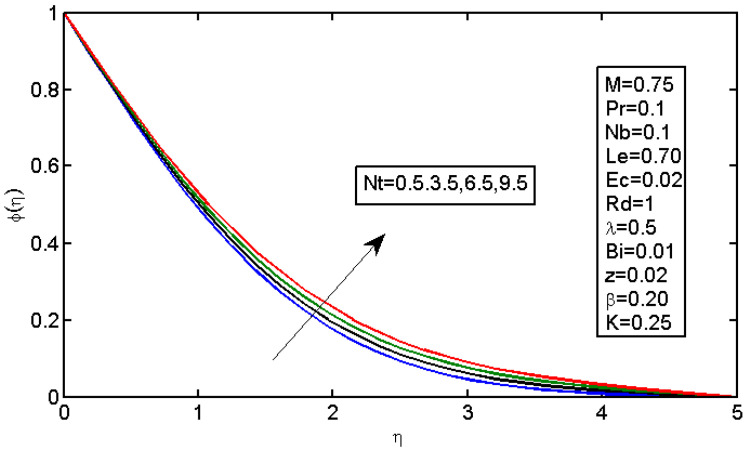
Impact of $N_{t}$ on the concentration profile.

**Figure 10:**
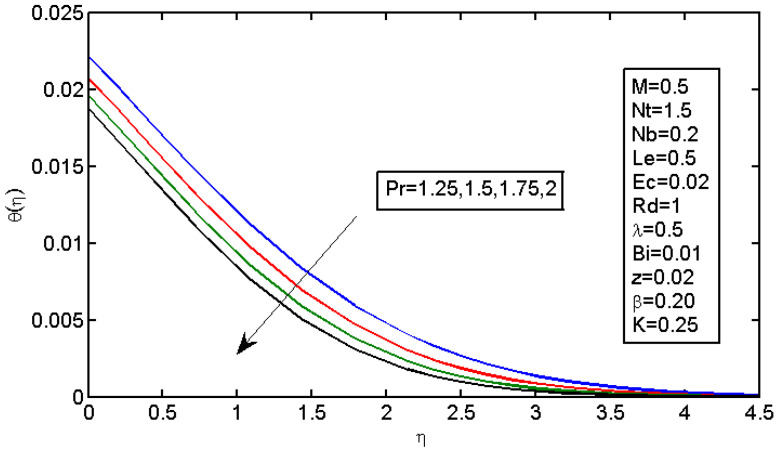
Impact of Pr on the temperature profile.

**Figure 11:**
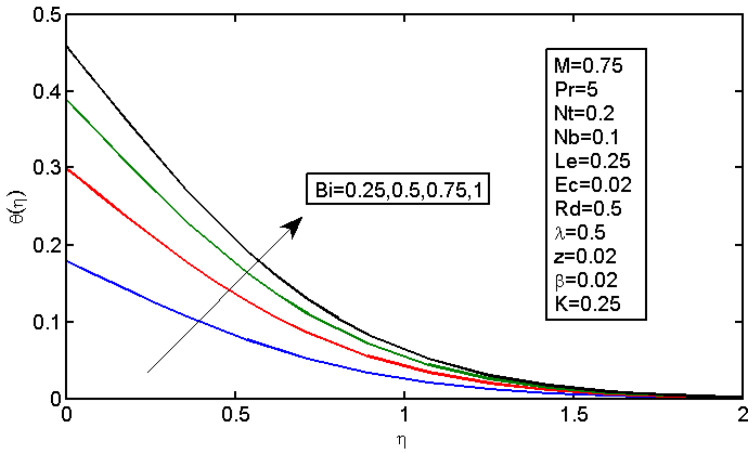
Impact of $B_{i}$ on the temperature profile.

**Figure 12:**
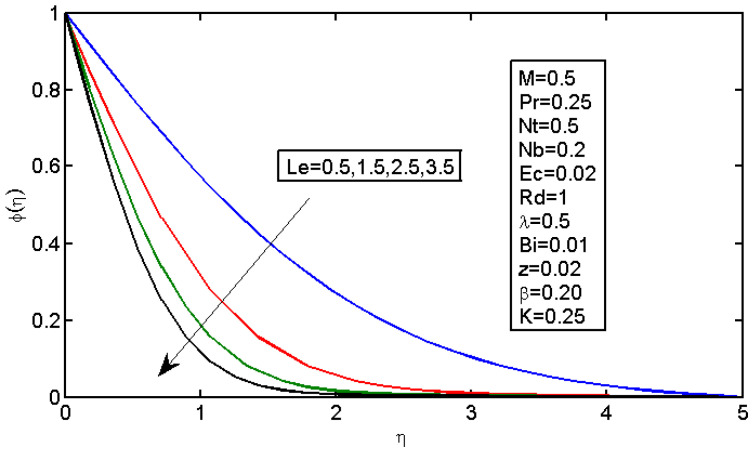
Impact of Le on the concentration profile.

A two-dimensional, steady boundary layer flow of MHD nanofluid through an extending sheet with the occurrence of thermal radiation was analyzed. We examined the graphical results of the problem from an actual perspective. The variations in speed, temperature and nanoparticles concentration with governing parameters, for example $N_{b}$, the parameter for Brownian motion; the Prandtl number $Pr,\;N_{t}$, the parameter for thermophoresis; the Biot number *B_i_* and the magnetic parameter (M), were determined using the Keller Box technique and the mathematical calculations were processed utilizing the MATLAB program. The outcomes are presented in Tables and the diagrams displayed in Figure 1, Figure 2, Figure 3, Figure 4, Figure 5, Figure 6, Figure 7, Figure 8, Figure 9, Figure 10, Figure 11 and Figure 12.

Figure 1 shows that the velocity in the boundary area was reduced by the slip parameter. Figure 2 shows that, by increasing the values of K, the velocity profile declined, i.e., with an increase in the value of K, the boundary layer thickness increased, so the velocity decreased with the increase in the value of K because the effect of the porous medium, which would act against the flow, also increased and enhanced the deceleration of the flow.

The results of magnetic field parameter M over the dimensionless velocity and temperature are displayed in Figure 3 and Figure 4, respectively. Figure 4 shows the effect of M on the velocity profile; it caused the velocity profile to decrease with a decrease in force, which resulted in a reduction in speed. In addition, higher values of M decreased the boundary layer thickness. In the Lorentz force, expansion was caused by the magnetic field; subsequently, the highly restricting power made it difficult for the liquid to stream without any hindrance. The magnetic field acted against the vehicle cycle. Figure 4 shows the impact of the magnetic field on the temperature profiles, which increased with an increase in M. The Lorentz force tended to increase the temperature in nanofluid movement. Thus, the thermal boundary layer thickness increased for a more grounded magnetic field. Figure 5 illustrates the impact of stretching boundary λ on the speed profile. It is apparent that expanding the upper side resulted in an increase in the speed field. However, expanding the upper side decreased the thickness of the force and limited the layers for both the non-Newtonian and Newtonian liquid cases.

Figure 6 and Figure 7 illustrate the impact of Brownian motion parameter $N_{b}$ on the temperature and nanoparticle volume concentration profile, respectively. Figure 6 presents the effects of different values of $N_{b}$ on the thermal boundary layer. At this phase, Brownian motion occurred due to the size of the nanoparticles in the nanofluids. In the transfer of heat, the motion of the particles in the fluid played a significant role. In the flow, the nanoparticles’ motion was affected by an increase in $N_{b}$. The temperature of the nanofluid increased when the kinetic energy of the nanoparticles was increased by the strength of this chaotic motion. Figure 7 illustrates the variations in the nanoparticle concentration with $N_{b}$. For stronger Brownian motion, a slight decrease in φ occurred accounted away from the sheet. The impact of thermophoresis parameter $N_{t}$ on the temperature profile and the concentration profile is presented in Figure 8 and Figure 9. Figure 8 illustrates the impact of the thermophoresis parameter of nanoparticles and thermal radiation on the thermal boundary layer. Additionally, it shows that the expansion in the thermophoresis boundary resulted in an increase in the thermophoretic force that transported nanoparticles from warm to cool spaces; however, the temperature profile increased due to this. Figure 9 shows the impact of $N_{t}$ on the nanoparticle concentration profile. With an increase in $N_{t}$, there was an increase in the wideness of the concentration boundary and overshoot formed near the wall. Figure 10 shows the impact of the Prandtl number on the nanofluid’s thermal boundaries. Practically, if Pr increased, there would be a decrease in the thermal diffusivity, and this principle would diminish the ability to transfer energy, decreasing the thermal boundary layer. Figure 11 depicts the impact of $B_{i}$ on the temperature profile. As the Biot number increased, the thermal boundary increased. Figure 12 illustrates the impact of *Le* on the nanoparticle concentration profile. At higher values of *Le*, the concentration of the nanoparticles greatly decreased. As Le increased, the thermal boundary layer thickness also increased; however, the thickness of the concentration boundary layer decreased.

Table 1 indicates that, for a weaker Brownian movement, $N_{b}=0.1$, the Nusselt number decreased with an increase in *N_t_*. As the Brownian motion increased (for example, $N_{b}$ changed from 0.1 to 0.4), the decrease in the Nusselt number became critical with an expansion in $N_{t}$. This is reasonable as the strengthened Brownian movement was comparable to the instant movement of nanoparticles that stretched the wall to the dormant fluid. The heat movement rate at the wall θ’(0) was an increasing function of the radiation boundary. It was additionally seen that, for a given $N_{b}$ value, the decrease in Nusselt number with $N_{t}$ was greater in the case of $R_{d}=0$ when compared with $R_{d}=1$. These observations were obtained from the results of Zeeshan [8]. Table 2 provides information for the decreased Sherwood number. The Sherwood number decreased when $N_{t}$ increased. This was because of the way that stronger thermophoresis impacts would generally move the nanoparticle volume fraction away from the surface, as the hot surface repulsed the sub-micron-sized particles, subsequently forming a moderately molecule-free layer close to the surface. For weaker Brownian movements and stronger Brownian movements, this decrease was constant.

## 7. Conclusions

This research is about the numerical examination of the impacts of thermal radiation on the MHD nanofluid flow when an extending sheet that is not linear is saturated in a permeable medium. The Keller Box method was used to obtain a numerical solution for governing non-linear equations after transformation by using similarity transformation. The mass and heat transfer rates, shear stress and other factors, such as the nanoparticle concentration profile, temperature and velocity, were computed numerically by using MATLAB, and their graphical representation is also provided. The MHD nanofluid flow was used to investigate the effects of slip with velocity when thermal radiation is applied. Some of the observations of interest in this study are

❖ The increase in the stretching sheet parameter suggests an increase in the velocity profile;
❖ The increase in the parameter of velocity slip decreased the velocity profile;
❖ The increase in the porosity parameter reduced the velocity profile;
❖ When the Brownian motion parameter increased, the nanoparticle concentration decreased, whereas the temperature increased;
❖ The nanoparticle concentration and temperature both increased with an increase in the thermophoresis parameter;
❖ By increasing the Prandtl number, the temperature profile decreased;
❖ The increase in the Biot number indicated an increase in the temperature profile;
❖ As Le increased, the concentration decreased;
❖ By increasing the Brownian motion parameter, the Sherwood number increased in contrast to the increase in the thermophoresis parameter;
❖ These results can be further extended for different non-Newtonian fluid models, such as Jeffrey fluid, viscoelastic fluid, couple stress fluid, Maxwell fluid, Oldroyd-B fluid, etc. Moreover, some other features can be discussed for current flow problems, such as entropy generation, heat source, activation energy, bioconvection, etc.

## Figures and Tables

**Table 1:** Values of the decreased Nusselt number $-\theta^{\prime }\left(0\right)$ when $Pr=7,\;M=0.5,\;\lambda =0.5,\;Ec=0.2,\;\theta_{w}=1.5,\;Le=10\;$ and $B_{i}=0.1$.

$N_{b}$	$R_{d}$	$N_{t}$			
		0.1	0.2	0.3	0.4
0.1	0 1	0.07543 0.3597	0.07418 0.3584	0.07271 0.3570	0.07095 0.3556
0.2	0 1	0.06623 0.3486	0.06365 0.3469	0.06042 0.3452	0.05623 0.3434
0.3	0 1	0.05249 0.3362	0.04712 0.3341	0.04013 0.3319	0.03023 0.3296
0.4	0 1	0.03307 0.3225	0.02328 0.3200	0.01018 0.3173	−0.006327 0.3145

**Table 2:** Values of the decreased Sherwood number $-\varphi^{\prime }\left(0\right)$ when $Pr=7,\;M=0.5,\;\lambda =0.5,\;Ec=0.2,\;\theta_{w}=1.5,\;Le=10\;$ and $B_{i}=0.1$.

$N_{b}$	$R_{d}$	$N_{t}$			
		0.1	0.2	0.3	0.4
0.1	0 1	2.43 2.37	2.535 2.396	2.65 2.424	2.783 2.456
0.2	0 1	2.417 2.364	2.5 2.384	2.597 2.406	2.714 2.429
0.3	0 1	2.418 2.363	2.504 2.381	2.609 2.401	2.737 2.422
0.4	0 1	2.424 2.362	2.518 2.380	2.630 2.399	2.756 2.419

## Data Availability

All the data are fully available in the manuscript.

## Nomenclature

a Stretching rate	T Fluid temperature
C Concentration	$T_{w}$ Surface temperature
$T_{\infty }$ Free stream temperature	$u_{\infty }$ Free stream velocity
(u,v) Velocity components	(x,y) Cartesian coordinates
$\sigma_{e}$ Electrically conductivity	$B_{0}$ Magnetic force strength
T Fluid temperature	C Fluid concentration
$D_{B}$ Brownian diffusion	$q_{r}$ Radiative flux
$v_{f}$ Fluid viscosity	α Thermal conductivity
L Slip factor	M Magnetic parameter
Rd Radiation parameter	β Slip parameter
Bi Biot number	Nb Brownian motion parameter
K Porosity parameter	λ Stretching parameter
Nt Thermophoresis parameter	Le Lewis number and
$E_{c}^{*}$ Local Eckert number	Pr Prandtl number
